# Evaluating the Effect of Body Mass Index on Procalcitonin Level in Patients with Pneumonia: A Retrospective Cross-Sectional Study

**DOI:** 10.3390/arm93010001

**Published:** 2025-01-26

**Authors:** Mohammad Z. Khrais, J. Curran Henson, Jake Smith, Nikhil Meena

**Affiliations:** 1 Department of Internal Medicine, University of Arkansas for Medical Sciences, Little Rock, AR 72205, USA; 2 Division of Pulmonary Medicine, University of Arkansas for Medical Sciences, Little Rock, AR 72205, USA

**Keywords:** procalcitonin, PCT, BMI, pneumonia, obesity

## Abstract

**Highlights:**

**What are the main findings?**

- Body mass index (BMI) has a statistically significant effect on serum procalcitonin (PCT) levels in hospitalized patients with pneumonia.
- Higher BMI patients exhibited significantly lower PCT levels compared to patients with lower BMI.

**What is the implication of the main finding?**

- The findings suggest that individuals with higher BMI might exhibit either a less robust immune response or increased PCT clearance.
- Consideration of BMI-adjusted PCT reference ranges could enhance diagnostic accuracy, though further research is needed to validate this approach.

**Abstract:**

Procalcitonin (PCT) is commonly used to evaluate the etiology and severity of pneumonia. PCT has been shown to be elevated at baseline in patients with obesity. The aim of this study is to determine if body mass index (BMI) has an influence on admission PCT level in patients admitted to the hospital with a diagnosis of pneumonia. We conducted a retrospective cohort study that encompassed patient visits to a tertiary care center from 2014 until September 2023. A total of 18,652 patients presented to the emergency department with a diagnosis of pneumonia. A total of 3659 were admitted to the floor, and 2246 were admitted to the MICU. Patients were grouped based on admission BMI into five categories. The Kruskal–Wallis test performed on patients admitted to the floor revealed a statistically significant difference in PCT levels among groups of different BMIs (H = 34.97, df = 4, *p* < 0.001). In patients admitted to the MICU, the Kruskal–Wallis test revealed a significant difference in PCT levels among groups of different BMIs (H = 32.92, df = 4, *p* < 0.001). BMI has a statistically significant effect on PCT in patients admitted to the hospital with pneumonia. Patients with higher BMI may exhibit less robust PCT levels, which may impact management decisions.

## 1. Introduction

Procalcitonin (PCT) and calcitonin gene-related peptide (CGRP) are transcriptional products of the calcitonin gene [1]. Both PCT and CGRP have been subjects of investigation regarding their relationship with body mass index (BMI) in various human and animal models [2,3]. It is plausible that PCT, synthesized in adipose tissue, may contribute to systemic inflammation in obesity, thus potentially explaining the observed correlation between higher BMI and elevated PCT levels [4].

PCT holds significant importance in clinical practice, particularly in the evaluation of pneumonia. Elevated PCT levels are indicative of bacterial infections, with concentrations of ≥0.5 μg/L having higher odds [5,6]. Both infectious and non-infectious factors, such as trauma, pancreatitis, certain cancers, and medications, can affect PCT levels [7]. The interpretation of PCT levels in the context of pneumonia diagnosis should be cautious, as elevated PCT levels have also been associated with many severe diseases; for instance, higher PCT levels were associated with severe COVID-19 infections [6,7]. This dual role of PCT underscores the importance of careful consideration when using PCT levels to guide clinical decisions regarding antibiotic therapy initiation or withholding.

Our study aims to investigate the relationship between PCT levels and BMI in a cohort of patients diagnosed with pneumonia. Additionally, we will explore PCT levels in subgroups of pneumonia patients categorized by their disposition, admitted to the hospital or admitted to the Medical Intensive Care Unit (MICU). Our research seeks to address the following questions: Could there be a relationship between PCT levels and BMI, independent of the disease etiology or severity?

## 2. Materials and Methods

This study consisted of a retrospective observational analysis to assess the association between procalcitonin levels and obesity classification in adult patients (Age ≥ 18 years) diagnosed with pneumonia in the ED of a tertiary medical center in the US. This study adheres to the Strengthening the Reporting of Observational Studies in Epidemiology (STROBE) guidelines, as outlined in Table S1 in the supplementary materials. Data were obtained using ICD diagnoses codes for pneumonia (J13, J14, J15.0, J15.1, J15.2, J15.3, J15.4, J15.5, J15.6, J15.7, J15.8, J15.9, J18.0, J18.1, J18.2, J18.8, J18.9) from 2014 to September 2023. Inclusion criteria encompassed cases with documented BMI and procalcitonin levels obtained within 48 h of admission. The collected data included BMI, procalcitonin levels, and patient disposition upon admission—whether they were admitted to the general medical floor of the hospital, or if they were ever admitted to the MICU during their hospitalization.

**Figure 1:**
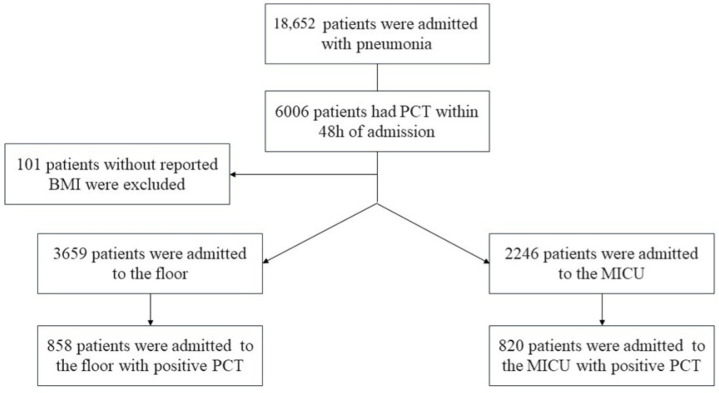
Algorithm of patient selection for the respective inclusive cohorts by admission disposition.

A total of 18,652 patients were admitted from the emergency department with a diagnosis of pneumonia [Figure 1]. A total of 6006 patients had a PCT level drawn within 48 h of admission, and 101 patients who did not have a reported BMI were excluded. A total of 3659 were admitted to the floor and 2246 were admitted to the MICU either on admission or during the same hospitalization [Figure 1]. Admitted patients were then further stratified by positive PCT test values (Positive PCT ≥ 0.5 defined by institutional standards). Duplicate PCT indices were subsequently removed for multiple BMI measurements of the same patients, resulting in cohorts of 858 and 820 patients with independent ‘positive’ PCT values admitted to the floor and MICU, respectively. Outlier analysis was performed using modified Z-score transformation, and secondary outlier elimination of variables not within 3.5 standard deviations of the median respective to the standard normal distribution BMI classification was performed with slight adjustments according to Centers for Disease Control and Prevention (CDC) classification into five different categories based on their associated BMI as follows: <20, 20–24.99, 25–29.99, 30–34.99, >35 [8]. This modification, not inclusive of the standard < 18.5 and 18.5–24.99 categories, was made to better equalize subgroup sizes and enhance the statistical balance of our analysis [8].

Univariate and multivariate linear regression models were performed to assess the individual and interaction relationships of BMI classification and admission disposition on patients with a confirmed diagnosis of pneumonia and a positive PCT value. Given the results identified in these models and given a non-normal distribution of the datasets with positive skew (only positive testing results included), non-parametric Kruskal–Wallis testing was first utilized to evaluate the effect of BMI on PCT for the respective individual cohorts followed by Dunn’s post hoc testing to evaluate intragroup comparisons. Given the results identified in these models and given a non-normal distribution of the datasets with positive skew (only positive testing results included), non-parametric Kruskal–Wallis testing was first utilized to evaluate the effect of BMI on PCT for the respective individual cohorts followed by Dunn’s post hoc non-parametric testing to evaluate intragroup comparisons.

## 3. Results

Independent univariate linear regression models revealed a statistically significant association of BMI on PCT levels *p* < 0.05, and hospital admission disposition *p* < 0.0001 when BMI > 35, with hospital disposition to the MICU used as the reference for all patients with a positive PCT value. When multivariate regression was performed, the overall significant effect of BMI was no longer observed, with a *p*-value of 0.062 when the same reference values were used. The multivariate model did yield significant differences between the different BMI and disposition classifications with BMI class [0–19.99] having the greatest statistically significant difference from the greatest BMI class [>35], *p* < 0.01, 95% CI 0.794 to 5.540, with a subsequent decrease in statistical difference for each interval increase in BMI class. Patient disposition status revealed a significantly lower PCT for patients admitted to the floor, *p* < 0.0001, 95% CI −5.896 to −3.349 [Table 1].

Given the potential interaction effects of admission status disposition on PCT levels, a cross-sectional analysis was conducted to determine the effects of BMI on PCT, dichotomized in a subgroup analysis based on patients’ respective hospital admission disposition status. Given the aforementioned non-parametric nature of the datasets, the Kruskal–Wallis test was utilized for all patients admitted to the floor, revealing a difference of (H = 34.97, df = 4, *p* < 0.001). Post hoc pairwise comparisons using Dunn’s test indicated that Group 1 (BMI < 20) significantly differed from Group 3 (BMI 25–29.99), Group 4 (BMI 30–34.99), and Group 5 (BMI > 35) (*p* = 0.009, <0.001, and <0.001, respectively). However, there was no significant difference between Group 1 and Group 2 (BMI 20–24.99) (*p* = 0.35). Similarly, Group 2 significantly differed from Group 3, Group 4, and Group 5 (*p* = 0.036, 0.001, and <0.001, respectively). Group 3 significantly differed from Group 5 (*p* = 0.005), but not from Group 4 (*p* = 0.18). No significant difference was observed between Group 4 and Group 5 (*p* = 0.19).

In all patients admitted to the MICU, the Kruskal–Wallis test revealed a significant difference in PCT levels among groups of different BMI (H = 32.92, df = 4, *p* < 0.001). Post hoc pairwise comparisons using Dunn’s test indicated that Group 5 (BMI > 35) significantly differed from all other groups; Group 1 (BMI < 20) (*p* < 0.001), Group 2 (BMI 20–24.99) (*p* < 0.001), Group 3 (BMI 25–29.99) (*p* < 0.001), and Group 4 (BMI 30–34.99) (*p* = 0.002).

**Figure 2:**
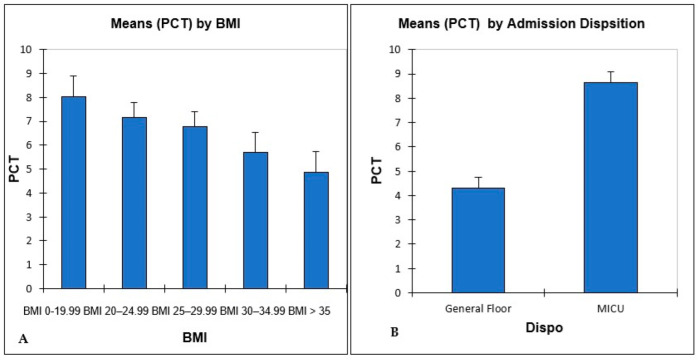
(**A**) Mean positive test PCT values by BMI classification +/− S.E for all patients included in linear regression models. (**B**) Mean positive test PCT value for all patients by respective hospital admission disposition, general floor (858), and MICU (820).

In both subgroups of patients with positive PCT levels, those admitted to the floor (858) and the MICU (820), the Kruskal–Wallis test did not reveal a significant difference in PCT levels among groups of different BMI, (H = 3.02, df = 4, *p* = 0.55) and (H = 6.16, df = 4, *p* = 0.19), respectively. However, an inverse relationship between BMI groups and mean Procalcitonin (PCT) levels was observed [Figure 2].

## 4. Discussion

Multiple studies have demonstrated a positive correlation between higher BMI and elevated PCT levels in normal subjects. This relationship is supported by the pro-inflammatory state associated with obesity and the fact that PCT is synthesized in adipose tissue. On the other hand, PCT, as an inflammatory marker, correlates with the severity of pneumonia [9]. These findings suggest that obese patients with pneumonia should have higher PCT levels, while lean patients exhibit a less robust reaction.

However, the relationship between PCT levels and BMI in disease states has produced mixed results. For example, a study of 105 critically ill patients with COVID pneumonia revealed no correlation between BMI and PCT levels [10]. In contrast, another study, in which obesity was identified as an independent risk factor for adverse outcomes and increased mortality in COVID patients, obese patients exhibited higher PCT levels [11]. Furthermore, Mentula et al. explored the relationship of BMI to inflammatory markers on admission in 117 subjects with acute pancreatitis, finding no correlation between PCT levels and BMI [12]. It is important to note that these studies, while informative, were limited by small sample sizes.

Our study revealed the opposite: a strong inverse correlation between PCT levels and higher BMI. These results may be explained by a more robust immune response in subjects with lower BMI or by a rapid immune response in this group of patients compared to their counterparts with higher BMI. In this instance, measurements of PCT clearance may have higher value, as this approach has been utilized to predict outcomes and serve as a prognostic biomarker in sepsis and septic shock [13]. Another theory explaining these results could be the increased PCT clearance in subjects with higher BMI. Like the observed inverse correlation between BMI and B-natriuretic peptide (BNP) levels, which is suggested to be due to increased expression of clearance receptors on adipose tissue in obese patients [14]. While this hypothesis is intriguing, it warrants further investigations, as it contradicts the higher baseline PCT levels reported in healthy obese individuals. It is possible that clearance mechanisms are altered during acute infections, or that the production–clearance balance shifts in favor of increased clearance during pneumonia in obese patients.

Another plausible explanation lies in the role of interferon (IFN) gamma. Previous studies have shown that PCT production is markedly reduced in adipose cell cultures, exposed to endotoxins, when co-administrated with IFN-gamma [15]. Obesity has been associated with elevated IFN-gamma expression [16], which could suppress PCT production. While this mechanism aligns with our findings, it does not explain the positive correlation between BMI and PCT levels in healthy individuals or the higher PCT levels observed in obese patients with COVID infection, where IFN-gamma plays a crucial role in the antiviral response. These apparent contradictions may reflect a shift in the type of immune response rather than an overall decrease in immune response in obese individuals. For example, obese patients with pneumonia may exhibit an immune response resembling that seen in viral infections due to elevated IFN-gamma levels. Alternatively, other immune modulators may influence PCT dynamics in this population. This underscores the complexity of the relationship between obesity, PCT levels, and different types of infections, highlighting the need for further research to clarify these interactions.

Our study has several limitations. Being a retrospective cross-sectional analysis, it is inherently limited by its design, which restricts the ability to infer causation. Additionally, reliance on ICD codes for data extraction may introduce misclassification bias. Potential confounders, such as age, illness severity, varying etiologies for pneumonia, kidney function, cancer diagnoses, and other factors, were not accounted for and may have influenced our findings. Future studies utilizing logistic regression models which incorporate these variables are essential to establish stronger correlations and more robust conclusions.

## 5. Conclusions

Patients admitted for pneumonia with a high BMI exhibited statistically significant lower levels of PCT compared to those with lower BMI. This observation suggests that individuals with higher BMI might develop a less robust or slower immune response, or they may demonstrate increased PCT clearance, resulting in lower serum PCT levels compared to patients with lower BMI. The consideration of BMI-adjusted reference ranges for PCT serum levels may be warranted but needs further study and validation. Future studies are essential to investigate serial PCT levels during hospitalization, in conjunction with other objective clinical evaluation tools and laboratory markers.

## Figures and Tables

**Table 1:** Model parameters of multivariate linear regression model with BMI > 35, and Hospital Dispo—MICU as reference categories.

Variable Category	Value	Standard Error	t	Pr > |t|	Lower Bound (95%)	Upper Bound (95%)	*p*-Value Signification Codes
Intercept	7.187	0.911	7.890	<0.0001	5.400	8.973	***
BMI-BMI 0–19.99	3.167	1.210	2.617	0.009	0.794	5.540	**
BMI-BMI 20–24.99	2.286	1.044	2.189	0.029	0.238	4.334	*
BMI-BMI 25–29.99	1.912	1.047	1.826	0.068	−0.142	3.965	.
BMI-BMI 30–34.99	0.837	1.190	0.703	0.482	−1.497	3.171	°
BMI-BMI > 35	0.000	0.000					
Hospital Dispo Gen Floor	−4.623	0.649	−7.120	<0.0001	−5.896	−3.349	***
Hospital Dispo MICU	0.000	0.000					

Signification codes: 0 < *** < 0.001 < ** < 0.01 < * < 0.05 < . < 0.1 < ° < 1.

## Data Availability

The data that support the findings of this study are available from the corresponding author upon reasonable request. Restrictions apply to the availability of these data, which were used under license for this study.

## Supplementary Materials

The following supporting information can be downloaded at: https://www.mdpi.com/article/10.3390/arm93010001/s1, Table S1: STROBE Statement.
